# Diversity of Cystathionine β-Synthase Haplotypes Bearing the Most Common Homocystinuria Mutation c.833T>C: A Possible Role for Gene Conversion

**DOI:** 10.1002/humu.20430

**Published:** 2006-10-27

**Authors:** Petr Vyletal, Jitka Sokolová, David N. Cooper, Jan P. Kraus, Michael Krawczak, Guglielmina Pepe, Olga Rickards, Hans G. Koch, Michael Linnebank, Leo A. J. Kluijtmans, Henk J. Blom, Godfried H. J. Boers, Mette Gaustadnes, Flemming Skovby, Bridget Wilcken, David E. L. Wilcken, Generoso Andria, Gianfranco Sebastio, Eileen R. Naughten, Sufin Yap, Toshihiro Ohura, Ewa Pronicka, Aranka Laszlo, Viktor Kožich

**Affiliations:** 1 Center for Applied Genomics, Institute of Inherited Metabolic Disorders, Charles University 1st Faculty of MedicinePrague, Czech Republic; 2 Institute of Medical Genetics, Cardiff UniversityCardiff, United Kingdom; 3 Department of Pediatrics, University of Colorado School of MedicineAurora, Colorado; 4 Institut für Medizinische Informatik und Statistik, Christian-Albrechts UniversitätKiel, Germany; 5 Department of Medical and Surgical Critical Care, Center of Research, Transfer, High Education “DENOthe,” University of FlorenceFlorence, Italy; 6 Centre of Molecular Anthropology for Ancient DNA Studies, Department of Biology, University of Rome “Tor Vergata,”Rome, Italy; 7 Department of Pediatrics, University Hospital MünsterMünster, Germany; 8 Department of Neurology, University Hospital BonnBonn, Germany; 9 Laboratory of Pediatrics and Neurology, University Medical Centre NijmegenThe Netherlands; 10 Department of Internal Medicine, University Hospital NijmegenNijmegen, The Netherlands; 11 Department of Clinical Biochemistry, Aarhus University HospitalAarhus, Denmark; 12 Department of Clinical Genetics, RigshospitaletCopenhagen, Denmark; 13 The Children's Hospital at WestmeadSydney, New South Wales, Australia; 14 Cardiovascular Genetics Laboratory, Prince of Wales HospitalRandwick, New South Wales, Australia; 15 Department of Paediatrics, Federico II UniversityNaples, Italy; 16 The National Centre of Inherited Metabolic Diseases, The Children's University HospitalDublin, Ireland; 17 Department of Pediatrics, Tohoku University School of MedicineSendai, Japan; 18 Division of Metabolic Diseases, Department of Paediatrics, Children's Memorial Health InstituteWarsaw, Poland; 19 Department of Pediatrics, Albert Szent-Gyorgyi Medical Center, University of SzegedSzeged, Hungary

**Keywords:** homocysteine, homocystinuria, haplotype, pyridoxal 5′phosphate, cystathionine beta-synthase, CBS, gene conversion

## Abstract

Homozygosity or compound heterozygosity for the c.833T>C transition (p.I278T) in the cystathionine beta-synthase (*CBS*) gene represents the most common cause of pyridoxine-responsive homocystinuria in Western Eurasians. However, the frequency of the pathogenic c.833C allele, as observed in healthy newborns from several European countries (q_c.833C_ ≊ 3.3 × 10^–3^), is ∼20-fold higher than expected on the basis of the observed number of symptomatic homocystinuria patients carrying this mutation (q_c.833C_ ≊ 0.18 × 10^–3^), implying clinical underascertainment. Intriguingly, the c.833C mutation is also present in combination with a 68-bp insertion, c.[833C; 844_845ins68], in a substantial proportion of chromosomes from nonhomocystinuric individuals worldwide. We have sought to study the relationship between the pathogenic and nonpathogenic c.833C-bearing chromosomes and to determine whether the pathogenic c.[833C; −] chromosomes are identical-by-descent or instead arose by recurrent mutation. Initial haplotype analysis of 780 randomly selected Czech and sub-Saharan African wild-type chromosomes, employing 12 intragenic markers, revealed 29 distinct *CBS* haplotypes, of which 10 carried the c.[833C; 844_845ins68] combination; none carried an isolated c.833C or c.844_845ins68 mutation. Subsequent examination of 69 pathogenic c.[833C; −] chromosomes, derived from homocystinuria patients of predominantly European origin, disclosed three unrelated haplotypes that differed from their wild-type counterparts by virtue of the presence of c.833C, thereby indicating that c.833T>C transition has occurred repeatedly and independently in the past. Since c.833T does not reside within an obvious mutational hotspot, we surmise that the three pathogenic and comparatively prevalent c.[833C; −] chromosomes may have originated by recurrent gene conversion employing the common nonpathogenic c.[833C; 844_845ins68] chromosomes as templates. Hum Mutat 28(3), 255–264, 2007. Published 2006 Wiley-Liss, Inc.†

## INTRODUCTION

Autosomal recessive cystathionine beta-synthase (CBS) deficiency (MIM# 236200) is the most common inborn error of sulfur metabolism and was first recognized over four decades ago [Mudd et al., 1964]. Untreated CBS deficiency is characterized clinically by skeletal and ocular abnormalities, as well as thromboembolism and mental retardation. However, early treatment by administration of high doses of pyridoxine and/or by methionine restriction combined with betaine administration has proven effective in preventing many complications of this disease [Mudd et al., 2001]. The clinical, biochemical, and molecular aspects of CBS deficiency have recently been reviewed in detail [Kožich and Kraus, 2001; Mudd et al., 2001].

Molecular analysis of the *CBS* gene in patients with homocystinuria has led to the identification of some 130 different pathogenic mutations, the majority of which are rare and private [Kraus et al., 1999] (www.hgmd.org; www.uchsc.edu/cbs/cbsdata/cgidata.htm). Although several mutations are frequent, only the transition c.833T>C (p.I278T) in exon 8 of the *CBS* gene [Kraus et al., 1999] has been reported to occur in virtually every studied population of European origin (www.uchsc.edu/cbs/cbsdata/cgidata.htm). The nonpolar Ile278 residue is highly conserved between mammals, *Neurospora* sp., *Anopheles gambiae, Pichia pastoris,* and several bacterial species. However, other species such as *Xenopus* sp., *Fugu rubripes, Drosophila melanogaster, Dictyostelium discoides, Caenorhabditis elegans,* and *Saccharomyces cerevisiae* have a smaller valine residue at the analogous position, suggesting that the enzyme may tolerate mild spatial alterations. By contrast, the substitution of the nonpolar isoleucine by the polar threonine is clearly unfavorable since the mutant enzyme is inactivated [Kožich and Kraus, 1992; Shan et al., 2001] as a consequence of misfolding, loss of heme, and protein aggregation [Janošík et al., 2001]. Despite the detrimental impact of the mutation on enzymatic properties, homozygosity or compound heterozygosity for the c.833C allele is consistently associated with a mild clinical phenotype, both in humans and in transgenic mice [Mudd et al., 2001; Wang et al., 2005]. More importantly, carriership of at least one c.833C allele in CBS-deficient patients confers clinical and biochemical responsiveness to vitamin B_6_ administration, and consequently necessitates a less severe therapeutic regimen [Kožich and Kraus, 2001; Mudd et al., 2001].

At present, the c.833C allele would appear to be the most common pathogenic *CBS* variant in Western Eurasians suffering from CBS deficiency. Indeed, in symptomatic homocystinuric patients, c.833C constitutes ∼25% of all homocystinuric alleles (132/553 alleles as of April 2006; www.uchsc.edu/cbs/cbsdata/cgidata.htm). Further, an unusually high prevalence of heterozygotes for this variant has been observed among Danish, German, Czech, and Norwegian newborns (i.e., 1:71, 1:67, 1:257, and 1:63, respectively) and in healthy Dutch adults (i.e., 1:250) [Gaustadnes et al., 1999; Griffioen et al., 2005; Linnebank et al., 2001a; Refsum et al., 2004; Sokolová et al., 2001].

**FIGURE 1:**
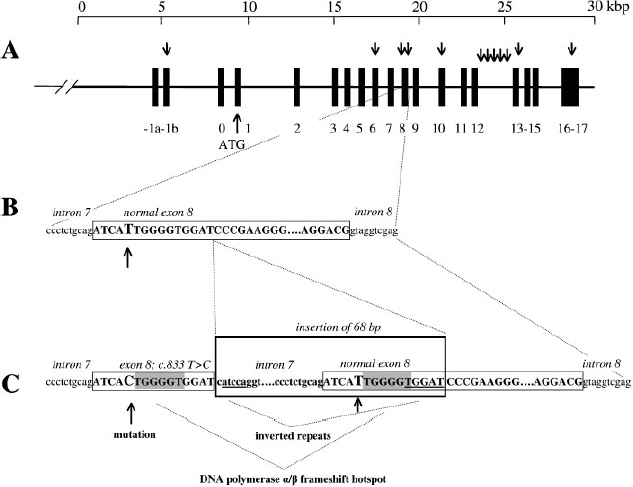
Structure of the wild-type c. [833T; –] and c. [833C; 844_845ins68] *CBS* chromosomes. **A**:The genomic organization of the *CBS* gene, with exons depicted as numbered black boxes and the initiator ATG in exon 1 indicated by an upward-pointing arrow. Markers genotyped in this study are denoted by downward-pointing arrows. **B**: Boxed uppercase letters represent the sequence of wild-type exon 8. The lowercase sequence corresponds to flanking intronic regions. The thymidine residue at nucleotide position 833 is marked by an arrow. **C**:The sequence of the variant c. [833C; 844_845ins68] chromosome. The 68-bp insertion between nucleotides 844 and 845 is bracketed. Both the mutation c.833C in exon 8 and the wild-type thymidine 833 within the 68-bp insertion sequence are indicated by arrows. Motifs corresponding to DNA polymerase α/β frameshift hotspots and inverted repeats are shaded and underlined, respectively.

Only a small proportion of human chromosomes carry the pathogenic mutation c.833C on its own (henceforth referred to as c.[833C; –] chromosomes), whereas a much larger proportion contain a nonpathogenic combination of two mutations (termed c.[833C; 844_845ins68] chromosomes). In the latter chromosomes, the pathogenic effect of c.833C is completely nullified by the downstream insertion of a 68-bp duplicated portion of the intron 7/exon 8 junction (Fig. 1C). Although this insertion creates two intron 7 splice donor sites in close proximity, the splicing machinery strongly favors the use of the more distal splice donor site, thereby removing the upstream segment of exon 8 together with the c.833C mutation while preserving the rest [Romano et al., 2002; Sperandeo et al., 1996b; Tsai et al., 1996]. Consequently, both heterozygotes and homozygotes carrying the nonpathogenic c. [833C; 844_845ins68] chromosomes synthesize normal *CBS* mRNA molecules lacking the pathogenic r.833U > C mutation; these individuals therefore exhibit neither biochemical nor clinical signs of homocystinuria [Pepe et al., 1999; Tsai et al., 1996]. The nonpathogenic c. [833C; 844_845ins68] chromosomes are very common in sub-Saharan Africa (up to 40% of control chromosomes), less frequent throughout Europe and America (5–10% of control chromosomes) [Franco et al., 1998b; Pepe et al., 1999], and comparatively rare in Asia (0.16–2.5% of control chromosomes) [Song et al., 2001; Zhang and Dai, 2001].

The relatively high frequency of pathogenic c. [833C; –] chromosomes in populations of European descent could have resulted from a number of different mechanisms, including recurrent mutation in a mutational hotspot or gene conversion using the common nonpathogenic c.[833C; 844_845ins68] chromosomes as templates. In addition, positive selection for carriers, random genetic drift or a combination of these mechanisms could have been responsible for boosting the population frequencies of the pathogenic c. [833C; –] chromosomes to their present values. We have adopted several different approaches in an attempt to distinguish between two competing hypotheses, namely: 1) that the pathogenic c.[833C; –] chromosomes are identical-by-descent; or 2) that these chromosomes arose by recurrent mutation, possibly involving c. [833C; 844_845ins68] chromosomes as templates.

## PATIENTS AND METHODS

### Patients

All patients in the present study suffered from homocystinuria due to CBS deficiency, and were either homozygous or compound heterozygous for the pathogenic c. [833C; –] chromosomes. Through an international cooperative effort, samples of genomic DNA, blood or fibroblasts were obtained from a total of 54 unrelated subjects, representing 71 independent c.[833C; –] chromosomes. A total of 11 patients each were from the Netherlands and Italy, nine were from Germany, six from Denmark, five from Australia, four from the Czech Republic, two each from Ireland and France, and one each from Poland, Hungary, Japan, and Slovakia. All subjects except the Japanese patient were of European ancestry.

Control individuals facilitating the haplotype analysis of wild-type *CBS* chromosomes had previously been ascertained in two different studies. Banked genomic DNA samples were obtained from 200 anonymous Czech [Janošíková et al., 2003] and 190 anonymous sub-Saharan African controls (50 subjects each from the Fon, Dendi, and Bariba populations, and 40 individuals from the Berba population) [Pepe et al., 1999]. All controls were apparently healthy individuals with no clinical cardiovascular or connective tissue manifestations.

The study protocol was approved by the Ethics Committee of Charles University-1st Faculty of Medicine. All homocystinuria patients gave their informed consent via their primary care physicians. Control subjects gave their written informed consent during the course of sampling in the original study.

### Genotyping

Genomic DNA was isolated from peripheral blood leukocytes or cultured human fibroblasts by column extraction. Genotypes for 12 polymorphic *CBS* markers (for overview, see Fig. 1A) were determined by fragment analysis of PCR products (C.1444_1467+7(16_21) [known as 31 bp variable number of tandem repeats (VNTR)]), amplification refractory mutations system PCR (ARMS-PCR) (c.699C>T, c.833T>C, c.844_845ins68, and C.1080C>T), PCR-RFLP (c.-3792G>A, c.699C>T, c.833T>C, c.1080C>T, and c.*543T>C [known as C.1985T>C]) and automated DNA sequencing (c.1358+161G>A, c.1358+264A>G, c. 1359–219C>T, c.1359–134G>A, and c.1359-30C>T). The ARMS-PCR and PCR-RFLP techniques were used interchangeably since they yielded identical results. For detailed conditions and suppliers of chemicals and instruments, see Supplementary Table S1 (available online at http://www.interscience.wiley.com/jpages/1059-7794/suppmat). The analysis of c.833T>C and c.844_845ins68 was designed to permit unequivocal phase determination of these two markers. The cDNA sequence numbering was based upon GenBank reference sequence NM_000071.1 (the first adenosine in the initiator ATG was designated as +1), genomic DNA numbering was based upon GenBank reference sequence NC_000021.7 and the numbering of the 31-bp VNTR was based upon a literature report [Lievers et al., 2001].

### Physical Haplotyping

A physical haplotyping method had to be developed in order to permit haplotype determination in all study subjects because no parental samples were available for phase determination. Since PCR amplification of a 20-kb segment of the *CBS* gene proved to be unreliable, PCR conditions to amplify a shorter 12.2-kb portion of the *CBS* gene between exons 5 and 17 were optimized and subsequently employed. Samples that yielded more than two haplotypes (probably due to PCR jumping [Pääbo et al., 1990]) and samples which failed in a 12.2-kb PCR amplification as a consequence of poor template DNA quality, were amplified in two overlapping 7.4-kb and 8.1-kb fragments spanning exon 5 to intron 13 and intron 9 to exon 17, respectively. Detailed conditions for the PCR reactions are given in Supplementary Table S1. The PCR products were gel-purified and cloned into a bacterial vector using the TOPO® XL PCR Cloning Kit (Invitrogen; www.invitrogen.com, Carlsbad, CA) according to the manufacturer's instructions. Individual alleles were then genotyped from plasmid DNA preparations as described above.

Throughout this work, the haplotypes have been described using the following reference haplotype: NM_000071.1: c.[-3792G>A; 699C>T; 833T>C; 844_845ins68; 1080C>T; intron 12 cluster; C.1444_1467+7(16_21); *543T>C]. The intron 12 variants c.1358+161G>A, c.1358+264A>G, c.1359-219C>T, c.1359-134G>A, and c.1359-30C>T were clustered as follows: α, [G; G; C; G; C]; β, [G; G; C; A; T]; γ, [G; G; T; A; T], and δ, [G; A; C; G; C].

### Haplotyping in Controls

Genotypes for seven polymorphic markers were first examined in all 390 control DNA samples, and HAPMAX (available at www.uni-kiel.de/medinfo/mitarbeiter/krawczak/download/index.html) analysis was performed to estimate the possible haplotype structure of control chromosomes. Next, we searched manually for homozygotes carrying the predicted haplotypes. In these samples, genotyping was extended to the remaining five markers by sequencing intron 12. If heterozygosity was observed for more than one locus in the latter analysis, physical haplotyping was employed. Several haplotypes predicted by HAPMAX were, however, individually rare and were not found in the homozygous state. Their existence was therefore also confirmed directly by the physical haplotyping of selected samples.

### Haplotyping in Patients Carrying Pathogenic c.[833C; –] Chromosomes

Genotypes for 12 polymorphic markers were determined in all genomic DNA samples from homocystinuria patients. Of the 54 samples available, two did not amplify in any PCR and seven failed in long-range PCR. The remaining 45 samples were amenable to genotyping and physical haplotyping. *CBS* haplotypes were determined either directly as a consequence of homozygosity for at least 11 out of 12 markers, or by physical haplotyping.

### Geographical Variation in c.[833C; –] Chromosome Frequency

For selected countries, we were able to estimate not only the population frequency of all homocystinuria-causing *CBS* alleles combined, but also the *CBS* alleles bearing the c.833T>C mutation. Since only a limited number of European countries perform long-term neonatal screening for CBS deficiency (and would thus have been able to provide reliable incidence data for deriving these estimates), we simply used the population prevalence of CBS deficiency to extrapolate allele frequencies. Data were first collected on the number of all known CBS-deficient patients and on their genotypes for each of the countries involved using a combination of three approaches: 1) a questionnaire-based survey, with 11 laboratories approached and eight replies received; 2) exploitation of the CBS Mutation Database (www.uchsc.edu/cbs/cbsdata/cgidata.htm); and 3) literature searches. Owing to its much greater ethnic heterogeneity, no attempt was made to perform this analysis in the United States. Population sizes were taken from the United Nations World Population Policies 2003 (www.un.org/esa/population/publications/wpp2003/Publication_index.htm). For crude data, see Supplementary Table S2. Assuming Hardy-Weinberg equilibrium, the total frequency of disease-causing *CBS* alleles was calculated as the square root of the prevalence of CBS deficiency. Then, the frequency of the c. [833C; –] chromosomes was estimated by multiplying this number by the proportion of c.[833C; –] chromosomes observed among patients. Finally, the combined population frequency of disease-causing CBS chromosomes in Europe was calculated from summary data on the numbers of patients and the sum of respective population sizes of these countries.

### Statistical Analyses

Maximum likelihood estimates of haplotype frequencies were obtained from genotype data of unrelated individuals using the HAPMAX computer program. Confidence intervals for the disease prevalence and allele frequency estimates were calculated using the Wilson score method without continuity correction [Newcombe, 1998].

## RESULTS

### Haplotype Analysis of Wild-Type CBS Chromosomes

First, we genotyped a set of seven polymorphic markers in all 390 control DNA samples from one European and four African populations; the African populations were included so as to provide the potential to acquire additional information on identical-by-descent c.833C-bearing chromosomes. Subsequently, five additional markers in intron 12 were genotyped in selected samples in order to determine their *CBS* haplotypes. The frequencies of most of the genotypes differed quite markedly between Czech and African controls (for details see Table 1). The frequencies of the c. 1080C>T and c.*543T>C variants were substantially higher in the Czech population whereas the frequency of the c. [833C; 844_845ins68] double mutation was considerably lower than in Africans. Importantly, we did not detect any c.833 Tchromosomes carrying only the c.844_845ins68 lesion (i.e., c.[833T; 844_845ins68]), nor did we detect any chromosomes bearing exclusively the c.833C mutation (i.e., c.[833C; –]). Thus, the c.844_845ins68 variant was in absolute linkage disequilibrium with the c.833C mutation in all population samples analyzed.

**TABLE 1:** *CBS* MarkerAllele Frequencies in Controls*

	Allele frequencies in different populations					
	
			African control populations			
			
Marker	Czech controls	African controls	Fon	Dendi	Berba	Bariba
Number of chromosomes	400	380	100	100	80	100
c.-3792G>A	0.01	0.00	0.00	0.00	0.00	0.00
c.699C>T	0.32	0.20	0.17	0.24	0.15	0.24
c.833T>C	0.07	0.33	0.33	0.30	0.36	0.24
c.844_845ins68	0.07	0.33	0.33	0.30	0.36	0.24
C.1080C>T	0.42	0.00	0.00	0.00	0.01	0.00
c.1444_1467+7(16)^a^	0.002	0.00	0.00	0.00	0.00	0.00
C.1444_1467+7(17)	0.07	0.22	0.29	0.15	0.24	0.21
C.1444_1467+7(18)	0.77	0.26	0.23	0.27	0.20	0.33
C.1444_1467+7(19)	0.11	0.19	0.12	0.16	0.15	0.13
C.1444_1467+7(21)	0.05	0.38	0.36	0.42	0.41	0.33
c.*543T>C (known as c.1985T>C)	0.46 (C)	0.12 (T)	0.14 (T)	0.14 (T)	0.13 (T)	0.07 (T)

* With the exception of the VNTR polymorphism, the marker frequencies are given as minor allele frequencies. The cDNA sequence numbering is based on GenBank reference sequence NM_000071.1 (the first adenosine in the initiator ATG is designated as + 1), genomic DNA numbering is based on GenBank reference sequence NC_000021.7 and the numbering of the 31-bp c.1444_1467+7(16.21) VNTR is based on a published report [Lievers et al., 2001]. For an overview of polymorphisms in the *CBS* gene, see [Kožich and Kraus, 2001].

a The length of the individual VNTR motifs is invariably 31 bp; however, different single-nucleotide substitutions may be present within the repeat units, as described previously by Lievers et al. [2001].

Next, haplotypes were constructed either from homozygous genotypes or by performing physical haplotyping, employing all 12 polymorphic markers. Haplotypes involving intron 12 markers c.1358+161, c.1358+264, c.1359–219, c.1359–134, and c.1359–30 could be divided into four different clusters, which we have termed α [G; G; C; G; C], β [G; G; C; A; T], γ [G; G; T; A; T], and δ [G; A; C; G; C], respectively.

Our study revealed a total of 29 and 18 different wild-type *CBS* haplotypes, respectively, depending upon whether or not the VNTR c.1444_1467+7(16_21) genotype was included (for details see Table 2). Chromosomes carrying the c.[833C; 844_845ins68] double mutation were found in both populations. European chromosomes were found to harbor only two or four haplotypes, respectively, depending upon whether or not VNTR heterogeneity was considered, while the ancestral African chromosomes displayed much greater haplotype variability. We attempted to compare the haplotype structure observed in our cohort with those generated by the HapMap Project (www.hapmap.org). Although HapMap release 19/phase II from October 2005 contains an array of *CBS* haplotypes, no direct comparison was possible owing to the use of a completely different set of *CBS* SNPs by the HapMap project.

**TABLE 2:** *CBS* Haplotypes Observed in Control Chromosomes

			Population^b^	
Haplotype^a^	Detection method	X, number of c.1444_1467+7(16_21) VNTR repeats	Czech	African
c.[833T;-] haplotypes				
[G; T; T; –; C; α; X; C]	P	21	−	+
[G; C; T; –; C; β X; C]	H	19	−	+
[G; T; T; –; C; β X; C]	H	19	−	+
[G; T; T; –; C; γ; X; C]	H	19	+	−
[G; T; T; –; C; γ; X; T]	H	18	+	+
same as above	H	19	+	+
[G; C; T; –; C; δ; X; C]	H	18	+	+
same as above	H	21	+	+
[G; C; T; –; C; δ; X; T]	H	17	+	+
same as above	H	18	+	+
same as above	H	19	+	+
same as above	H	21	+	+
[G; T; T; –; C; δ; X; T]	H	17	+	+
same as above	H	18	+	+
same as above	H	21	+	+
[G; C; T; –; T; δ; X; C]	H	18	+	−
[G; T; T; –; T; δ; X; C]	P	16	+	−
same as above	H	18	+	−
[G; C; T; –; T; δ; X; T]	H	18	+	−
c.[833C; 844_845ins68] haplotypes				
[G; C; C; ins; C; α; X; C]	P	18	−	+
[G; C; C; ins; C; β; X; T]	H	17	+	+
same as above	H	18	+	+
same as above	H	21	+	+
[A; C; C; ins; C; β; X; T]	P	17	+	−
[G; C; C; ins; T; β; X; T]	P	17	−	+
[G; T; C; ins; C; β; X; T]	H	17	−	+
[G; C; C; ins; C; δ; X; C]	P	21	−	+
[G; C; C; ins; C; δ; X; T]	H	17	−	+
same as above	H	19	−	1

Haplotypes were determined either by homozygosity of at least 11 markers (detection method H) or by physical haplotyping (detection method P).

a Haplotypes are described in relation to the reference *CBS* haplotype- NM_000071.1: c.[-3792G>A; 699C>T; 833T>C; 844_845ins68; 1080C>T; intron 12 cluster; c.1444_1467+7(16_21); *543T>C], for details see Patients and Methods. The intron 12 variants C.1358+161G>A, c.1358 + 264A>G, c.1359-219C>T, c.1359-134G>A and c.1359-30C>T were clustered as follows: α, [G; G; C; G; C]; β, [G; G; C; A;T]; γ [G; G; T; A; T] and δ, [G; A; C; G; C].

b +, haplotype observed in the studied population sample; −, haplotype not detected in the studied population sample.

### Population Frequency and Haplotype Structure of Homocystinuric Chromosomes

The population frequency of patients with clinically ascertained and biochemically confirmed CBS deficiency varied considerably between countries, averaging ∼1 out of 1,500,000 inhabitants in Europe and yielding an estimated population frequency of pathogenic *CBS* alleles of 0.82 × 10^−3^. The population frequency of the c.833C allele, as estimated from the number of homocystinuria patients carrying at least one c.[833C; –] chromosome, varied by an order of magnitude between the 13 European countries, with the highest prevalence being observed in northern Europe. The calculated frequency of this allele in Europe (q_c.833C_ ≊ 0.18 × 10^−3^) was ∼20-fold lower than actually observed in healthy controls—mostly unselected newborns—from several European countries (q_c.833C_ ≊ 3.3 × 10^−3^) (see Table 3). This discrepancy is suggestive either of the decreased antenatal viability of c. [833C; –] homozygotes, the premature death of patients, or, rather more plausibly, an ascertainment bias with respect to patients having a clinically less apparent and milder pyridoxine-responsive form of the disease due to c.[833C; –] homozygosity.

**TABLE 3:** Population Frequency of Pathogenic *CBS* Chromosomes*

	Frequency calculated from number of diagnosed patients with homocystinuria^a^		Frequency determined in healthy controls^b^
		
Country	All pathogenic *CBS* chromosomes	c. [833C; –] chromosomes	c. [833C; –] chromosomes
Czech Republic	1.1 (0.86–1.5)	0.30 (0.23–0.40)	2.0 (0.83; 4.6)
Denmark	1.6 (1.2–2.1)	0.81 (0.62–1.0)	7.0 (3.4–14.6)
France	0.52 (0.40–0.66)	0.048 (0.038–0.062)	
Germany	0.76 (0.66–0.88)	0.21 (0.19–0.25)	7.5 (2.6–22.0)
Hungary	1.1 (0.83–1.5)	0.046 (0.035–0.061)	
Ireland	3.3 (2.8–3.8)	0.12 (0.099–0.13)	
Italy	0.58 (0.46–0.72)	0.14 (0.11–0.17)	
Norway	1.5 (1.1–2.0)	0.15 (0.11–0.20)	3.1 (1.5–6.4)
Netherlands	1.5 (1.2–1.7)	0.80 (0.68–0.94)	2.0 (0.5–7.3)
Poland	0.66 (0.52–0.84)	0.020 (0.015–0.025)	
Slovakia	1.2 (0.87–1.7)	0.076 (0.054–0.11)	
Spain	0.58 (0.45–0.76)	0 (0.0–0.0)	
United Kingdom	0.49 (0.38–0.63)	0.14 (0.11–0.18)	
Europe	0.82 (0.77–0.87)	0.18 (0.17–0.19)	3.3 (2.2–5.0)
Australia-New South Wales	2.4 (2.1–2.9)	0.31 (0.26–0.36)	0 (0.0–4.0)
Japan	0.58 (0.50–0.67)	0.013 (0.012–0.016)	

* Frequencies are given as number per 1,000 chromosomes (i.e., x 10 ^−3^).

a Raw data for calculating allele frequencies are given in Supplementary Table S2; the calculations are described in detail in the Patients and Methods section.

b Population frequencies of the c.[833C; –] chromosome were calculated from original data published for Czech [Sokolová et al., 2001], Danish [Gaustadnes et al., 1999], German [Linnebank et al., 2001a], Norwegian [Refsum et al., 2004], and Australian newborns (personal communication by B. Wilcken; n = 1,160 consecutive newborns), and for Dutch healthy controls [Griffioen et al., 2005]. The 95% confidence intervals are given in parentheses.

Haplotypes of c.[833C; –] chromosomes and their frequencies were determined by a combination of physical haplotyping and statistical analysis. Physical haplotyping of two-thirds of the available mutant chromosomes revealed three distinct and unrelated haplotypes ([G; C; C; –; C; δ; 21; T], [G; C; C; –; T; δ; 18; C], and [G; T; C; –; C; δ 19; C]) that differ from their respective wild-type counterparts only by the substitution of c.833C for c.833T. To reveal additional haplotypes that might have been present in samples that were not amenable to physical haplotyping, a maximum likelihood analysis was performed on the genotypes of 52 homocystinuric patients (representative of 69 c.[833C; –] chromosomes). As is evident from Supplementary Table S3, HAPMAX revealed the same three c.[833C; –] haplotypes as before, with frequency estimates that were virtually identical to those determined by physical haplotyping. Interestingly, the haplotype containing the δ cluster of intron 12 variants has been previously observed in patients of German origin (designated the “A1 haplotype” in Linnebank et al. [2001b]). We were unable to detect any c.833C-bearing haplotype containing cluster β of intron 12 variants (designated the “B1 haplotype” in the Linnebank et al. [2001b] study), and with hindsight this may have been due to different although partially overlapping sets of German patients having been included in the original and the present study.

**FIGURE 2:**
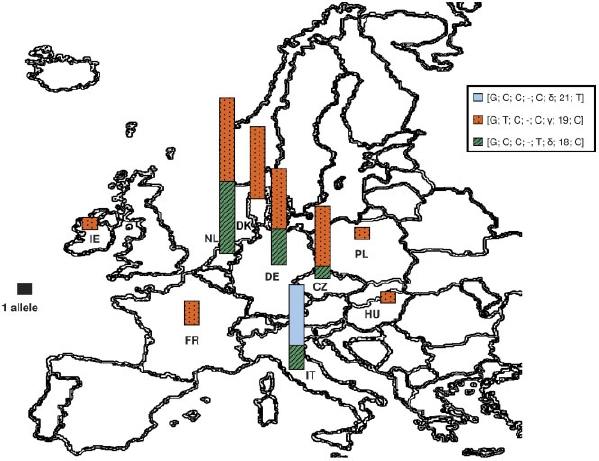
Distribution of c.[833C; –] haplotypes in Europe.Three different pathogenic haplotypes ([G; C; C; –; C; δ; 21;T], [G; C; C; –;T; δ; 18; C], and [G; T; C; –; C; γ; 19; C]) were observed among European homocystinuria patients. Haplotypes are described in relation to the reference *CBS* haplotype, for details see Patients and Methods. The number of chromosomes is proportional to the size of the column in each country, normalized by the black square representing one allele; it may also be found in Supplementary Table S3. [Color figure can be viewed in the online issue, which is available at www.interscience.wiley.com.]

The distribution of different pathogenic c. [833C; –] haplotypes varied between different European countries (see Fig. 2), reflecting the complex population history of the mutant *CBS* chromosomes. It is important to stress that we did not observe any heterogeneity in the number of VNTR repeats for any of the mutant haplotypes, consistent with a rather recent occurrence of all mutant c. [833C; –] chromosomes. The [G; T; C; –; C; γ; 19; C] haplotype was present in 8 out of 9 European countries tested, suggesting that it might be the most ancient one. By contrast, [G; C; C; –; T; δ; 18; C] was restricted to the northern/central part of Europe, while [G; C; C; –; C; δ; 21; T] was confined to Italy. This variable geographical distribution suggests either different migration patterns or the more recent occurrence of the latter two mutant haplotypes.

### Search for Mutational Hotspots in the Vicinity of c.833T

There is no obvious reason to suppose that thymidine in position c.833 is hypermutable per se. Moreover, the immediate vicinity of the nucleotide c.833 does not contain any repetitive sequence elements that could have templated the c.833T>C transition and hence accounted for its independent occurrence on distinct haplotypes.

However, analysis of the regions flanking the adjacent nucleotide c.844, at which the 68-bp duplication occurs, revealed the presence of two sequence elements with mutational potential (see Fig. 1C). The first, at position c.834_839, was a 5′-TGGGGT-3′ sequence that matches the DNA polymerase α/β frameshift hotspot consensus sequence 5′-TG(G/A)(A/G)(G/A)(C/T)-3′ [Abeysinghe et al., 2003]. The second was an inverted repeat ATCCA/TGGAT, flanking the 68-bp insertion site that appears to be capable of forming an imperfect hairpin loop that could have mediated the exclusion of the inserted sequence while still preserving the pathogenic c.833C mutation on the same chromosome.

In summary, analysis of mutational hotspots and the haplotypes of c.833C-bearing chromosomes suggests a complex history for their generation, with neutral c. [833C; 844_845ins68] chromosomes having potentially templated the generation of mutant c.[833C; –] chromosomes via several mechanisms (see Discussion).

## DISCUSSION

In this study, we have presented evidence to support the view that the most common homocystinuria mutation c.833T>C (p.I278T) occurred both repeatedly and independently during the recent history of European populations. This assertion is based mainly upon the observation of three unrelated *CBS* haplotypes containing solely the pathogenic c.833T>C substitution. Since the recurrence of the c.833T>C mutation cannot be explained by any known mutational hotspot in the vicinity of c.833T, a different mutational mechanism should be considered.

The high prevalence of the neutral c.[833C; 844_845ins68] chromosomes in Europe prompted us to propose that these nonpathogenic chromosomes may have templated the repeated generation of pathogenic c. [833C; –] chromosomes. Different mutational mechanisms such as meiotic recombination, loop formation with subsequent excision, or gene conversion could have been responsible for recurrently converting wild-type chromosomes into pathogenic c.[833C; –] ones, using neutral c. [833C; 844_845ins68] chromosomes as templates.

Meiotic recombination as a mechanism for repeated mutagenesis appears unlikely owing to the close physical proximity of nucleotides 833 and 844. A meiotic event should have generated a c.844_845ins68 chromosome lacking the c.833C mutation. In 15 published studies containing data on the phase of the c.833C mutation and the c.844_845ins68 variant, a total of 10,074 *CBS* chromosomes were genotyped and 1,721 c. [833C; 844_845ins68] chromosomes were found. However, not a single c.833 T chromosome carrying only the c.844_845ins68 variant (i.e., c.[833T; 844_845ins68]) has been reported, indicating complete linkage disequilibrium of the c.844_845ins68 variant with c.833C [Aras et al., 2000; Dilley et al., 2001; Dutta et al., 2005; Fillon-Emery et al., 2004; Franco et al., 1998a, 1998b; Giusti et al., 1999; Griffioen et al., 2005; Janošíková et al., 2003; Orendáč et al., 1999; Pepe et al., 1999; Sokolová et al., 2001; Tsai et al., 2000, 1999; Zoossmann-Diskin et al., 2004].

The inverted repeat flanking the 68-bp insertion could, however, have templated the recurrent conversion of neutral c. [833C; 844_845ins68] chromosomes into pathogenic c.[833C; –] chromosomes via loop formation and excision. To explore this second hypothesis, we compared both the pathogenic and neutral haplotypes harboring c.833C. The two major European pathogenic haplotypes, i.e., [G; T; C; –; C; γ; 19; C] and [G; C; C; –; T; δ; 18; C], are unrelated to any of the neutral c.[833C; 844_845ins68] haplotypes. Therefore, their emergence by loop excision from any of the known neutral c.[833C; 844_845ins68] chromosomes would appear to be inherently unlikely. By contrast, the rare pathogenic haplotype [G; C; C; –; C; δ; 21; T] differs from the nonpathogenic haplotype [G; C; C; ins; C; δ; 17/19; T] only by virtue of the absence of the insertion at position c.844_845 and by the number of VNTR repeats. A complex, albeit still feasible, mechanism involving both loop excision and DNA polymerase slippage in the VNTR locus could have led to the formation of pathogenic [G; C; C; –; C; δ; 21; T] chromosomes. However, the putatively templating chromosome was only found in sub-Saharan African controls while the corresponding pathogenic haplotype was only present in Italy.

**FIGURE 3:**
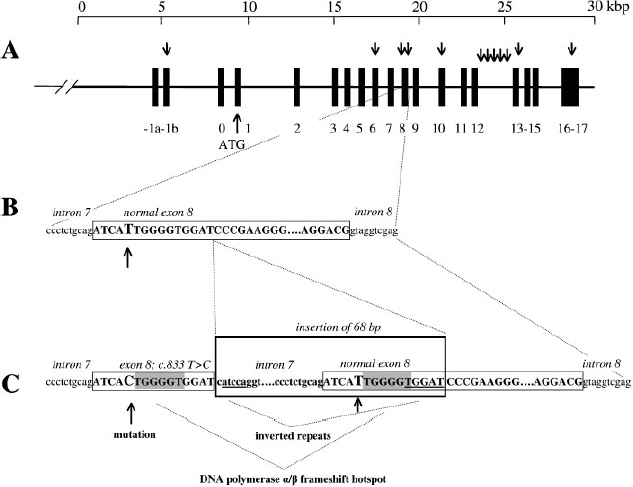
Gene conversion as a possible mechanism for recurrent mutation. A hypothetical mechanism by which the c. [833C; 844_845ins68] chromosomes could serve as template for the generation of the pathogenic c. [833C; –] chromosomes. The upper two strands represent the wild-type c.[833T; –] chromosomes and the two bottom strands represent a variant wild-type c. [833C; 844_845ins68] chromosome. Intron 7 is shown as a black line, exon 8 is a gray line, and the insertion of 68 bp is shown in green. The pathogenic mutation c.833C is shown as a red square. In step A, a double strand break occurs at the DNA polymerase α/β frameshift hotspot followed by 5′→3′ exonuclease activity on the c.[833T; –] chromosome in step B. Subsequent strand invasion occurs in step C and results in the formation of a Holliday junction in step D. In step E, strandrepair leads to the occurrence of a loop that is subsequently cleaved, generating the neutral c. [833C; 844_845ins68] and pathogenic c.[833C; –] chromosomes during gametogenesis (step F).

The third hypothesis invoking gene conversion, however, is favored by the observation that the c.833T>C mutation abuts the DNA polymerase α/β frameshift hotspot consensus sequence 5′-TG(G/A)(A/G)(G/A)(C/T)-3′, known to be involved in strand breakage. Moreover, all three mutant c. [833C; –] haplotypes have a wild-type c. [833T; –] counterpart, consistent with their emergence via gene conversion. In addition, the VNTR heterogeneity characteristic of the non-pathogenic c. [833C; 844_845ins68] chromosomes is lacking on mutant c. [833C; –] chromosomes. This is consistent with the view that the neutral c. [833C; 844_845ins68] chromosomes preceded the mutant c. [833C; –] chromosomes in evolutionary time. We therefore propose that, in individuals heterozygous for the wild-type c.[833T; –] and c. [833C; 844_845ins68] chromosomes, a double-strand break of the c. [833T; –] chromosome at the DNA polymerase α/β frameshift hotspot consensus sequence may have occurred. Subsequent strand invasion, formation of Holliday junctions and excision repair of an incomplete loop could then have formed the three mutant c.[833C, –] haplotypes (the proposed mechanism is depicted in Fig. 3). Gene conversion has been implicated in a number of different human diseases [Patrinos and Grosveld, 2003] and we propose that homocystinuria due to the c.833T>C transition may be yet another example.

A wide variability in the frequency of pathogenic c.[833C; –] chromosomes obtained from known homocystinuria patients in different populations is evident; northern Europe has the highest frequency while Japan has the lowest and the Iberian peninsula appears to be entirely devoid of c. [833C; –] chromosomes. If the nonpathogenic c.[833C; 844_845ins68] chromosomes did indeed template the creation of mutant c.[833C; –] chromosomes by gene conversion, one would expect that the population frequency of these mutant chromosomes would be positively correlated with the prevalence of the nonpathogenic ones. Consistent with this prediction, the low prevalence of c.[833C; 844_845ins68] chromosomes in Asia is indeed associated with the low prevalence, or even absence, of the c. [833C; –] chromosomes among Japanese [Katsushima et al., 2006] and Korean [Lee et al., 2005] homocystinuria patients, whereas a much higher prevalence of both the pathogenic and neutral chromosomes is apparent in Europe. Unfortunately, the assessment of this correlation could not be extended to sub-Saharan Africa due to a lack of reliable data on the frequency of homocystinuria in countries from this region, and because the control sample size in our study was probably too small to detect c.[833C; –]+[833T; –] heterozygotes.

It is at present unclear how much of the variability in the prevalence of the pathogenic c. [833C; –] chromosome can be attributed to the above mentioned mutagenic mechanisms as opposed to random drift, migration, and selection. It is also unclear to what extent this variability is due to the negative clinical ascertainment bias of mildly affected patients carrying the pathogenic c. [833C; –] chromosome (homozygotes and compound heterozygotes for this allelic variant manifest a pyridoxine-responsive homocystinuria with a milder clinical phenotype and such individuals may have not invariably been diagnosed as having CBS deficiency). This possibility is supported by an increasing number of reports of c.[833C; –]+[833C; –] homozygotes and of c. [833C; –]+[other] compound heterozygotes who suffer from an unusual form of the disease, manifesting only a thrombotic diathesis without affecting connective tissue or the central nervous system [Gaustadnes et al., 2000, 2002; Linnebank et al., 2003; Maclean et al., 2002].

The phenotypic expression of the c.833T>C mutation in heterozygotes is essentially unknown [Guttormsen et al., 2001; Sperandeo et al., 1996a]. It is also unclear whether females heterozygous for the pathogenic c.[833C; –] chromosomes may have had a more favorable pregnancy outcome (reduced bleeding potential)—as proposed in cases of thrombophilia [Gopel et al., 2001; Lindqvist et al., 1998]—under the as yet unproven assumption that heterozygotes for CBS deficiency are in general more prone to thromboembolism [Mudd et al., 1981; Swift and Morrell, 1982]. It remains possible that the general living conditions could have been more disadvantageous to heterozygotes in Africa than in Europe, so that the question of whether or not the high frequency of c.833C in Europe may have been in part due to selection remains open.

Our study has revealed a considerable haplotype diversity of wild-type *CBS* chromosomes in the studied European and African populations. Were the VNTR polymorphism also to be considered, the number of haplotypes common to both populations would be 14. Czech controls carried six population-specific haplotypes, whereas controls from Africa exhibited nine unique haplotypes. These findings are consistent with the more recent emergence of European populations as compared to the ancestral populations originating in Africa.

In conclusion, our study has demonstrated significant worldwide differences in the frequency of disease-causing c. [833C; –] chromosomes, which are associated with three unrelated *CBS* haplotypes in populations of predominantly European origin. We propose that a complex evolutionary process was responsible for the formation of these haplotypes and that the common neutral c. [833C; 844_845ins68] chromosomes might have been a source of pathogenic c.[833C; –] chromosomes by mechanisms that probably involved gene conversion.
